# Separation of Highly Pure Semiconducting Single-Wall Carbon Nanotubes in Alkane Solvents via Double Liquid-Phase Extraction

**DOI:** 10.3390/nano15010023

**Published:** 2024-12-27

**Authors:** Ahmad Al Shboul, Mohamed Siaj, Jerome Claverie

**Affiliations:** 1 Department of Electrical Engineering, École de Technologie Supérieure ÉTS, 1100 Notre-Dame St. W, Montreal, QC H3C 1K3, Canada; 2 Department of Chemical Engineering and Biotechnology Engineering, University of Sherbrooke, 2500, Boul. de l’Université, Sherbrooke, QC J1K 2R1, Canada; 3 Department of Chemistry, University of Sherbrooke, 2500, Blvd de l’Université, Sherbrooke, QC J1K 2R1, Canada

**Keywords:** cholesterol-based polymer, double liquid-phase extraction (DLPE), direct liquid-phase exfoliation (DLPE), partition, semiconducting, metallic, single-wall carbon nanotubes (SWCNTs)

## Abstract

This study delves into the distinctive selective property exhibited by a non-conjugated cholesterol-based polymer, poly(CEM_11_-*b*-EHA_7_), in sorting semiconducting single-walled carbon nanotubes (s-SWCNTs) within isooctane. Comprised of 11 repeating units of cholesteryloxycarbonyl-2-hydroxy methacrylate (CEM) and 7 repeating units of 2-ethylhexyl acrylate (EHA), this non-conjugated polymer demonstrates robust supramolecular interactions across the sp^2^ surface structure of carbon nanotubes and graphene. When coupled with the Double Liquid-Phase Extraction (DLPE) technology, the polymer effectively segregates s-SWCNTs into the isooctane phase (nonpolar) while excluding metallic SWCNTs (m-SWCNTs) in the water phase (polar). DLPE proves particularly efficient in partitioning larger-diameter s-SWCNTs (0.85–1.0 nm) compared to those dispersed directly in isooctane by poly(CEM_11_-*b*-EHA_7_) using direct liquid-phase exfoliation (LPE) techniques for diameters ranging from 0.75 to 0.95 nm. The DLPE method, bolstered by poly(CEM_11_-*b*-EHA_7_), successfully eliminates impurities from s-SWCNT extraction, including residual metallic catalysts and carbonaceous substances, which constitute up to 20% of raw HiPCO SWCNTs. DLPE emerges as a scalable and straightforward approach for selectively extracting s-SWCNTs in nonpolar, low-boiling-point solvents like alkanes. These dispersions hold promise for fabricating fast-drying s-SWCNT inks, which are ideal for printed and flexible thin-film transistors.

## 1. Introduction

Single-walled carbon nanotubes (SWCNTs) have remarkable properties that have durably impacted the world of nanotechnology [1,2]. SWCNTs are increasingly used for the EMI shielding of composites in electronic and medical devices and the aerospace and defense sectors [3]. However, their synthesis yields heterogeneous mixtures of uneven purity that later impact electronic applications. SWCNTs are synthesized as heterogeneous mixtures of aggregated bundles with varying chiralities, diameters, and electronic types (i.e., metallic (m-SWCNTs) and semiconducting (s-SWCNTs)) [1]. This issue has attracted substantial attention since the utility of SWCNTs is limited in the absence of appropriate techniques for sorting them [1,4,5].

Various purification and sorting techniques have been proposed, such as dielectrophoresis [6], density gradient ultracentrifugation (DGU) [7], chromatography [8], DNA [9], and aqueous two-phase extraction (ATPE) [10,11,12]. Chen and Nish pioneered the fluorene-based polymers that develop π-π interactions with s-SWCNTs sidewalls [13,14]. Conjugated polymers have since been investigated as efficient tools for the large-scale extraction of s-SWCNTs in supernatants while m-SWCNTs precipitate at the bottom of the dispersion [1,3,4,15]. Conjugated polymers have also effectively sorted nanotubes based on their lengths [16,17] and diameters [18,19,20]. Notably, polymer properties such as rigidity [21], conformation [20,21,22], molecular weights (Mw) [23], conjugated structure surface area units within polymers’ backbone [24], and side-chain length [25,26] can all play an important role in sorting nanotubes.

Prior research has shown that aligned CNT arrays can be assembled for high-performance electronics applications and that conjugated polymers can be used for CNT separation. Shi et al. [27], Liu et al. [28], and Cheng et al. [29], for instance, were able to create aligned CNT-based field-effect transistors (FETs) with remarkable electronic characteristics and proved that they performed better than silicon devices. However, these approaches necessitate lengthy manufacturing stages and mostly concentrate on alignment-based strategies.

Nonetheless, when using s-SWCNT wrapped in conjugated polymers, charge transfer can occur between the conjugated polymer structure and the nanotube sidewalls, decreasing the e/hole mobility and the on/off ratio in transistors [30,31]. To solve this issue, techniques for releasing conjugated polymers after partitioning were developed, such as annealing [32], employing isomerized polymer [33,34], or using a polymer exchange technique with a favorable polymer with lower bandgap energy [35]. Solvent properties and solubility parameters can also considerably impact polymer selectivity, hence s-SWCNT partitioning [19,20,21,23,36]. Furthermore, solvents with strong dipole moments can interfere with charge transfer between polymers and SWCNTs, making polymers less selective [37,38]. As a result, a successful partition of SWCNTs necessitates using the proper solvents.

Wang et al. [37] described appropriate solvents that can solubilize the polymer and cannot disperse SWCNTs without the polymer. They must have a lower density than SWCNTs to facilitate precipitation in the unexfoliated tubes. Low-polar aromatic solvents such as toluene have been widely employed in partitioning s-SWCNTs by conjugated polymers [36,39]. Such aromatic solvent was found inadequate to separate large-diameter nanotubes, and it can cause quenching of the electronic transport characteristics in the inter-tube network [40,41].

This paper reports the sorting of s-SWCNTs in alkane solvents using a non-conjugated cholesterol-based polymer, poly(CEM_11_-*b*-EHA_7_), comprised of 11 repeating units of cholesteryloxycarbonyl-2-hydroxymethacrylate (CEM) and 7 repeating units of 2-ethylhexyl acrylate (EHA). The polymer’s design leverages cholesterol’s high adsorption energy over carbon materials, such as carbon nanotubes (CNTs) [42], pristine graphene [43], and reduced graphene oxide (RGO) [44], which are high (E_ad_ = −62 kJ·mol^−1^). In poly(CEM_11_-*b*-EHA_7_), the CEM groups adsorb at the surface of the carbon materials, while the EHA groups remain free in the solvent and create a hairy layer that sterically stabilizes the SWCNT. Compared to conjugated polymers, wrapping cholesterol-based polymers around nanotube renders any charge transfer to polymer impossible due to the non-conjugated saturated alkane nature of the cholesterol unit.

The coated nanotubes, sterically stabilized with cholesterol-based polymer in isooctane, effectively minimize aggregation and promote nanotube individualization in the dispersion. In contrast, uncoated nanotubes, lacking the protective cholesterol polymer, precipitate readily due to isooctane’s physical properties, hydrophobic nature, favorable Hansen, and solubility parameters (HSPs), facilitating efficient separation.

Isooctane has low viscosity (0.47 cP) [45] and density (0.7 g·mL^−1^) [45,46], making it an ideal solvent for the separation process, as it facilitates the exclusion of the aggregated nanotubes from the dispersion without the need for centrifugation, thereby streamlining the separation process and simplifying the overall technique. Furthermore, isooctane is very hydrophobic, and the dispersive (δ_D_), polar (δ_P_), and H-bond (δ_H_) of HSPs are, respectively, δ_D_ = 14–17, δ_P_ = 0, and δ_H_ = 0 MPa^1/2^ [47]. The combination of cholesterol polymer and isooctane forms a powerful strategy to prevent SWCNT aggregation, ensuring a streamlined, stable, and perfectly dispersed system.

**Figure 1:**
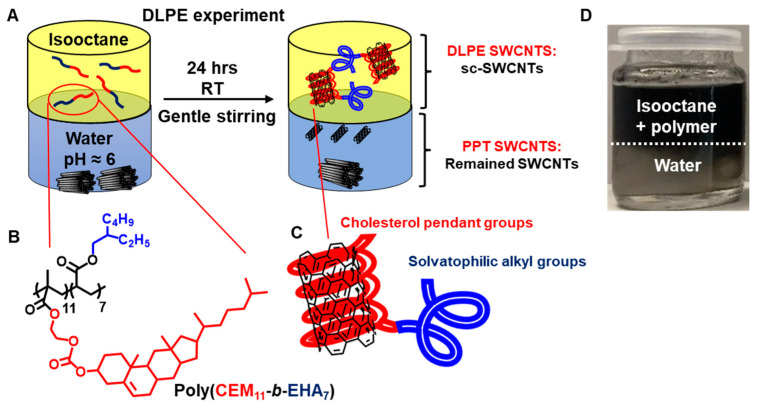
(**A**) Schematic illustration of the DLPE experiment. Raw HiPCO SWCNTs are covered with water and isooctane dissolving poly(CEM_11_-*b*-EHA_7_) (CEM = cholesteryloxycarbonyl-2-hydroxy methacrylate; EHA = 2-ethylhexyl acrylate). (**B**) Structure of the diblock copolymer poly(CEM_11_-*b*-EHA_7_). (**C**) Schematic illustration of wrapping the cholesterol groups around sidewalls of SWCNTs, while alkyl groups extending in the isooctane solvent responsible for creating a layer to stabilize nanotubes sterically. (**D**) Photograph the biphasic DLPE medium, showing the aqueous phase at the bottom (pH = 6) and isooctane phase containing the cholesterol-based polymer (0.25 mg·mL^−1^) after 12 h of gentle stirring at room temperature.

Thus, being able to disperse s-SWCNTs in an alkane solvent has allowed us to selectively extract larger-diameter nanotubes, making it a novel approach toward the purification of large-diameter nanotubes. This work uses a Double Liquid-Phase Extraction (DLPE) technique to sort s-SWCNTs in a single step (Figure 1A). This system comprises two immiscible phases: a nonpolar alkane solvent and polar water. Isooctane was chosen as an alkane because its structure resembles the polymer’s solvatophilic component, the ethyl-hexyl group (Figure 1B) [43,44].

## 2. Materials and Methods

### 2.1. Materials

HiPCO SWCNTs (Batch HPR 26-019), nanotubes produced through the high-pressure carbon monoxide process, were obtained from NanoIntegris. The synthesis and characterization of the block copolymer poly(CEM_11_-*b*-EHA_7_) (Figure 1B) were reported previously [42,44]. 2-hydroxyethyl methacrylate, 2-ethylhexyl acrylate (EHA), sodium dodecyl sulfonate (SDS), 1,4-dioxane, dimethyl sulfoxide (DMF), deuterium oxide (D_2_O), and isooctane were received from Sigma-Aldrich. Cholesteryl chloroformate was purchased from Alfa Aesar. Deionized water (resistivity > 18 MΩ·cm^−1^) was used for all experiments. All chemicals were used as received without further purification.

### 2.2. DLPE and LPE Techniques for SWCNT Dispersion

All experiments were performed by stirring the raw HiPCO SWCNT powder (1 mg). In the DLPE technique, while the lower phase is nanopure water (10 mL), the upper phase is isooctane (10 mL) dissolving poly(CEM_11_-*b*-EHA_7_) (c = 0.25 mg·mL^−1^). The system was continually stirred at 1000 rpm with a magnetic stir bar. After 12 h, a homogeneous SWCNT dispersion in the isooctane phase was separated from the aqueous phase using a Pasteur pipette. The precipitate of SWCNTs continued floating at the interface of the isooctane and water phases. The operational settings ([polymer] = 0.25 mg·mL^−1^, pH = 6, and room temperature) were discovered to be the ideal operating parameters for partitioning RGO by DLPE based on the quality of the flakes [44]. Therefore, they were used again to partition SWCNTs. While the SWCNTs extracted in the isooctane phase were labeled DLPE SWCNTs, the precipitate was labeled ppt DLPE.

To confirm DLPE technique efficiency, a comparison was established with a control SWCNT dispersion generated directly by liquid-phase exfoliation (LPE) process in isooctane with 0.25 mg·mL^−1^ of poly(CEM_11_-*b*-EHA_7_), the same polymer concentration utilized in the DLPE technique. The LPE SWCNT sample was ultrasonically dispersed for 10 min using a sonic dismembrator (Fisher Scientific, Hampton, NH, USA), Model 500, with a 0.5 in. diameter tip operating at 30 W to create a well-dispersed mixture, then stirred for 12 h on a magnetic bar. After the stirring was stopped, all samples were left for an hour to settle any aggregated or unbounded SWCNTs in the dispersion. Supernatants were gathered without any further processing. The dispersion of SWCNTs was labeled as LPE SWCNTs, while the non-exfoliated nanotubes (the precipitate) were labeled ppt LPE.

### 2.3. Preparation of Control Dispersions of SWCNTs in DMF and Deuterium Oxide

Control dispersions were prepared in DMF and D_2_O. First, 1 mg of raw HiPCO nanotubes was ultrasonicated for 10 min in 10 mL of DMF using a sonic dismembrator to create a well-dispersed mixture, and then the dispersion was stirred for 12 h on a magnetic bar. After the stirring was stopped, the dispersion was centrifuged for 1 h at 3500 rpm to precipitate colloidal contaminants and nanotube aggregations. DMF is generally considered an effective solvent for dispersing and individualizing SWCNTs, offering good dispersibility and stable dispersions without additional surfactants [48]. However, DMF is expected to disperse all the sample components, including nanotube impurities such as residual metallic catalysts and carbonaceous compounds. The control dispersion in DMF was labeled as DMF/SWCNTs. Alternately, 1 mg of raw SWCNTs were mixed with 1 mg·mL^−1^ of sodium dodecyl sulfate (SDS) dissolved in D_2_O solution. The liquid was then sonicated for 10 min using a sonic dismembrator before being stirred for 12 h over a magnetic bar stirrer. These dispersions were centrifuged for 1 h at 3500 rpm to precipitate colloidal contaminants, nanotube aggregations, or unbounded SWCNTs in the dispersion. Supernatants were collected, characterized, and compared to DLPE SWCNTs and LPE SWCNT samples. The SWCNT dispersion in SDS/D_2_O solution was labeled as SWCNTs/SDS.

### 2.4. Characterization

The SWCNT dispersion samples with a 0.01 mg·mL^−1^ concentration were analyzed using a model NS2 NanoSpectralyzer (Applied NanoFluorescence, Houston, TX, USA) for Visible/Near Infrared (Vis-NIR) absorption, Raman, and photoluminescence (PL) spectroscopy. Absorption spectroscopy was obtained in the 400–1600 nm region using a quartz cuvette and a spectral resolution of 1 nm interval (1 cm path length). SWCNT absorbance spectrum is classified into three sections. The first (S11) and second (S22) s-SWCNT transitions are identified at around 1000–1600 and 600–1000 nm, respectively. At 400–600 nm, the first transition of m-SWCNTs (M11) is observed [49,50].

Raman spectroscopy samples were placed in quartz cuvettes, and spectra were taken at 671 nm excitation laser wavelength. For each sample, all measurements were taken with an exposure period of 10,000 ms and an integration time of 500 acquisitions. The Radial Breathing Mode (RBM) in Raman spectroscopy offers valuable information on the m- and s-SWCNTs ratios in the dispersion. Nonetheless, critical aspects should be considered in the RBM. First, the excitation wavelength overlaps differently with chirality and diameter size. While small-diameter nanotubes and m-SWCNTs are resonant at 533 nm excitation wavelengths, large-diameter nanotubes and s-SWCNTs are resonant at 785 nm excitation wavelengths. Different diameter sizes and electronic types of SWCNTs (m- and s-SWCNTs) are resonant with intermediate excitation wavelengths as long as 633 nm [50,51]. The excitation wavelength in the NS2 NanoSpectralyzer is limited to 671 nm.

PL was carried out at three excitation wavelengths (532, 638, and 671 nm), and emission spectra were obtained in the 900–1400 nm range across 250 acquisitions using a 1000 ms integration period. PL is an effective chirality-based characterization technique for identifying s-SWCNTs, whereas m-SWCNTs are not detectable. The temperatures of material decomposition were determined using thermogravimetric analysis (TGA Q500 from TA Instruments, New Castle, DE, USA) with a heating rate of 10 °C·min^−1^ and an airflow rate of 100 mL·min^−1^. The microstructure of the samples was examined using transmission electron microscopy (TEM, JEOL JEM-2100F, Japan) equipped with a field emission gun operating at 200 kV. TEM samples were prepared by dipping Lacey grids (TED PELLA, Redding, CA, USA) in the SWCNT dispersion and allowed to dry for 10 min before measurements. Isooctane evaporates entirely in the air after 2 min at ambient temperature [43].

## 3. Results and Discussion

The DLPE technique presents two immiscible phases: isooctane (nonpolar or hydrophobic phase) and nanopure water (polar or hydrophilic phase). Figure 1A depicts the partitioning technique for SWCNTs. In pure isooctane, graphene and carbon nanotubes are not dispersible [42,43,44]. They become dispersible in isooctane in the presence of poly(CEM_11_-*b*-EHA_7_) (structure depicted in Figure 1B) as supramolecular interactions (E_ad_ = −62 kJ·mol^−1^) emerge between graphene structures and cholesterol units [42,43,44]. Interestingly, our past work demonstrated that block copolymers of CEM and EHA offer the best affinity for carbon nanotubes (MWCNT and SWCNT) compared to random copolymers and star copolymers [42].

Previously, DLPE was successfully employed to sort RGO based on the quality of its heterogeneous mixture [44], which had flakes that differed by their oxygenated defects generated during the graphene oxide (GO) reduction stage. In short, GO containing fewer defects is more hydrophobic and easier to disperse in the alkane phase. SWCNTs, however, are employed unoxidized, and one does not expect to observe significant variations in the surface concentration of oxygenated defects within a batch of unsorted SWCNTs. However, the hydrophobicity of nanotubes varies in two unique regimes depending on their diameter and electrical type [52]. The hydrophobicity of SWCNT increases with its diameter and reaches a plateau for diameters greater than 1 nm [52]. Hydrophobicity also depends on metallicity in the diameter regime greater than 1.2 nm (>1.2 nm) [52]. As a result, the s-SWCNTs with the largest diameter are the most hydrophobic, while smaller diameters of s-SWCNTs and m-SWCNTs are polarizable. Based on the observation, DLPE can initiate nanotube partitioning based on their hydrophobicity, most likely large-diameter s-SWCNTs, while excluding polarizable nanotubes, most likely small-diameter s-SWCNTs and m-SWCNTs [53,54].

In the DLPE experiment, HiPCO SWCNT powder is submerged in a water phase, followed by a phase of isooctane containing 0.25 mg·mL^−1^ of poly (CEM_11_-*b*-EHA_7_) (the polymer is only soluble in the organic phase). The organic phase was removed after stirring the biphasic mixture for 12 h (Figure 1C). The organic phase containing the extracted DLPE SWCNT remained colloidally stable over days (Figure 1D). The left-over aqueous phase contained undispersed aggregates of SWCNTs (ppt DLPE SWCNTs) floating at the surface, and there was a low concentration of dispersed SWCNTs.

The partitioning mechanism hinges on the cholesterol adsorption-release dynamics on the sidewalls of SWCNTs. When raw HiPCO SWCNTs are mixed with DLPE, the cholesterol-based polymer is expected to wrap around the sidewalls of the nanotubes and slowly extract them to the organic phase, resulting in a polymer/SWCNT dispersion in isooctane (Figure 1C). As previously observed, the cholesterol-based polymer is expected to be released when the polymer-wrapped nanotubes encounter water [43,44]. The released polymer will dissolve again in isooctane, wrapping other available nanotubes. The release and adsorption processes result in the separation of SWCNTs from the aqueous phase to the organic phase (isooctane) until an equilibrium distribution is reached. Thus, a successful DLPE experiment requires a sufficiently long contact time (12 h) between both phases, and experiments with shorter contact times failed in our hands. The resulting equilibrium distribution of cholesterol-based polymer binding affinity toward nanotubes and the characteristics of the nanotubes (hydrophobicity, diameter size) are all factors that can influence such final distribution.

We also have dispersed HiPCO SWCNTs using the direct LPE process for comparison. In such a process, the SWCNTs are covered by a solution of poly(CEM_11_-*b*-EHA_7_) polymer in isooctane (c = 0.25 mg·mL^−1^), and the mixture is sonication for 10 min using a sonic dismembrator and left under gentle stirring for 12 h. The dispersion (LPE SWCNTs) is then separated from the precipitated SWCNTs. Note that no sonication is necessary during the DLPE experiment. Indeed, due to the high adsorption energy of the cholesteryl unit [42,43,44], it was found that poly (CEM_11_-*b*-EHA_7_) polymer was able to disperse RGO without the need for sonication in a DLPE experiment. Here, a similar behavior is observed for carbon nanotubes. It should, however, be pointed out that only a small fraction of the SWCNTs (around 20% based on optical absorption) is extracted by the DLPE experiment.

**Figure 2:**
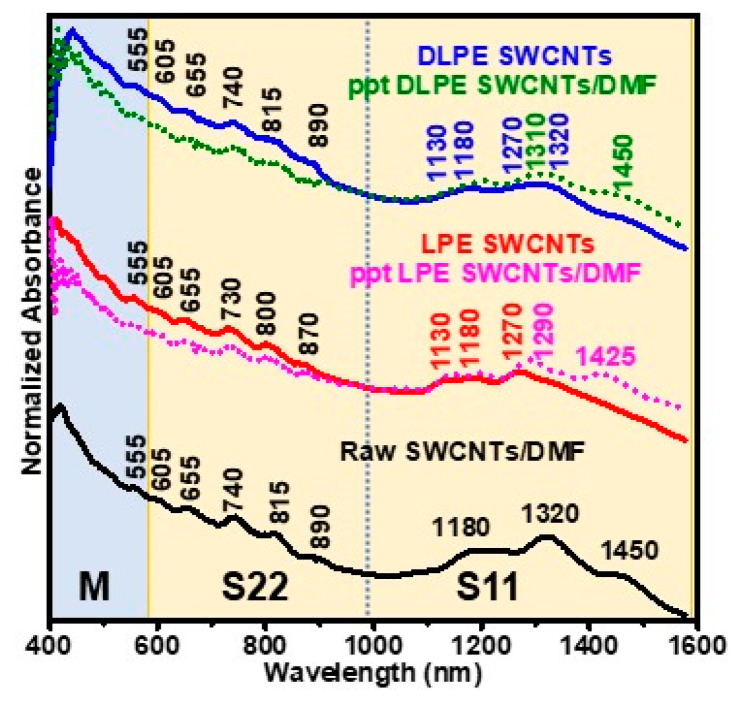
Vis-NIR absorption spectra for the control dispersion of HiPCO SWNCTs in DMF (SWCNTs/DMF), LPE SWCNTs, DLPE SWCNTs, and the precipitates from LPE (ppt LPE SWCNTs) and DLPE system (ppt DLPE SWCNTs) are indicated by dotted lines. Absorbance spectra are intensity normalized at 984 nm.

Figure 2 depicts the Vis-NIR absorption spectra for a series of SWCNT dispersions fixed at a 0.01 mg mL^−1^ concentration to prevent aggregation and minimize turbidity-induced effects, which occur when suspended nanoparticles scatter light instead of absorption [55]. However, an elevation in the background spectra rather than a change in the feature of the absorption spectra can be observed due to turbidity-induced effects [55]. Still, diluted SWCNT dispersions were applied to reduce nanoparticle interactions and aggregation, thus decreasing turbidity, improving transparency, and maintaining a stable dispersion. All absorption spectra were normalized at 984 nm [56], the wavelength between the S11 and S22 areas.

The absorption spectrum of the control dispersion of raw HiPCO SWCNTs in DMF shows three prominent peaks in the S11 region, located at 1450, 1320, and 1180 nm. Furthermore, it displays a sequence of minor peaks in the S22 region at 890, 815, 740, 655, and 605 nm. The LPE SWCNTs revealed that the absorption spectra shrank, and the S11 region blue-shifted to three peaks at 1270, 1180, and 1130 nm. At the same time, the peaks in the S22 area blue-shifted by 10–20 nm. The attenuation of the Vis-NIR absorption spectra hints at the ability of the cholesterol-based polymer to extract s-SWCNTs selectively.

The S11 area of the DLPE SWCNTs absorption spectrum was further altered by decreasing the peaks at 1270 nm and 1130 nm. As a result, two broad peaks can be seen at 1270–1320 nm and 1130–1180 nm. The S22 peaks in the DLPE SWCNTs have similar positions to those of raw SWCNTs/DMF dispersion. The absorption spectra in the precipitates (ppt LPE and ppt DLPE) demonstrated a close match one of raw SWCNTs/DMF, which can indicate the presence of the excluded SWCNTs from LPE SWCNTs and DLPE SWCNT dispersions.

With these supramolecular polymer/SWCNTs complexes, analyzing the M11 transition spectra is difficult. The M11 transition spectra of LPE and DLPE SWCNTs display absorption spectra identical to the control dispersion in DMF. This phenomenon can be attributed to various factors, including the π-plasmon of residual sample impurities [13], formulations of SWCNT bundles, or m-SWCNT presence [57]. Therefore, quantitatively assessing the purity of m-/s-SWCNT dispersions by studying absorption spectra alone is complicated.

Noteworthy, a similar absorption background was seen for HiPCO SWCNTs when sorted only by fluorene-based aromatic polymers [13] or by the ATPE technique [58,59]. Using CoMoCAT SWCNTs instead of HiPCO SWCNTs, on the other hand, the background absorption was eliminated, producing significant absorption spectra [13]. The change in the absorption spectrum indicates the importance of the SWCNTs’ source in producing ideal absorption spectra. Therefore, evaluating the efficiency of SWCNT individualization and separation based solely on absorbance techniques can be complex, and additional methods, as will be shown later, should be employed to validate the success of the process.

The broad absorption spectra for LPE and DLPE, rather than suggesting poor dispersion (low SWCNT individualization), may reflect the distinct interactions between the solvent, nanotubes, and polymer. The cholesterol-based polymer coating can induce strain or conformational changes in the SWCNT structure due to the high adsorption energy of cholesterol over the sp^2^ carbon framework (E_ad_ = −62 kJ·mol^−1^). The complex interplay between the SWCNTs and the polymer dispersant likely generates changes in the dielectric environment around the SWCNTs without disrupting their π-conjugated systems, modifying their band structure and exciton dynamics and contributing to the observed spectral signature. Similar influences have been discussed for SWCNTs wrapped with conjugated polymers [60]. Further investigation into these interactions could provide valuable insights into this intriguing spectral behavior.

Likewise, for the DMF dispersion, a similar phenomenon could be explained by considering DMF’s strong interaction with the SWCNT surface [61]. DMF is known to be a good solvent for SWCNTs due to its ability to form strong van der Waals interactions with the nanotube walls [62]. These interactions could potentially modify the electronic structure of the SWCNTs and may also contribute to this spectral behavior [62]. In both cases, the low absorption spectra could result from the specific solvent-SWCNT or polymer-SWCNT interactions, as well as the characteristics of the SWCNT source, rather than necessarily indicating poor dispersion or re-aggregation.

Alternatively, Ding et al. [40] proposed utilizing Raman spectroscopy to confirm s-SWCNTs’ purity. The Raman spectra of SWCNTs exhibit several characteristic features that provide valuable information about their structure and properties [63]. The main spectral elements include the RBM, typically observed between 100 and 350 cm^−1^, which is unique to indicate SWCNT electronic type and inversely related to tube diameter. The D-band, located around 1350 cm^−1^, indicates the presence of defects in the carbon lattice. The G-band, found at approximately 1580–1600 cm^−1^, is a hallmark of graphitic materials. The G′-band (also called 2D-band) appears around 2600–2700 cm^−1^ and is always present, even in defect-free samples.

**Figure 3:**
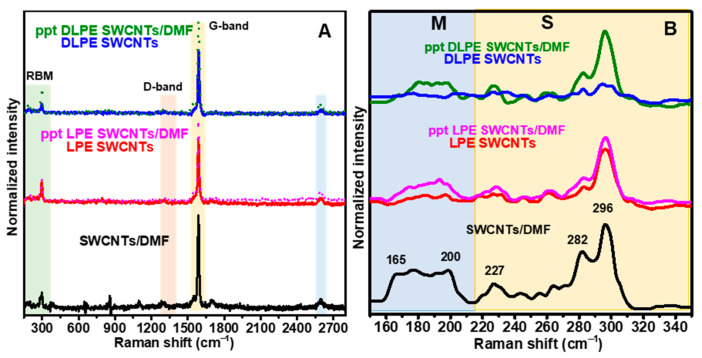
(**A**) Full Raman spectra and (**B**) RBM for dispersions of HiPCO SWNCTs in DMF (SWCNTs/DMF), LPE SWCNTs, DLPE SWCNTs, and their precipitates (ppt LPE SWCNTs and ppt DLPE SWCNTs).

Figure 3A shows full Raman spectra for dispersions of HiPCO SWNCTs. The strong G-band confirms the presence of a well-preserved graphitic structure, while the absence of the D-band indicates minimal structural defects. As discussed earlier, the low G’-band intensity may be attributed to the unique distinct electronic environment around the SWCNTs, potentially altering their Raman scattering behavior rather than indicating poor dispersion or quality. The polymer coating likely shields SWCNTs from environmental quenchers while minimizing non-radiative recombination pathways [64]. To avoid this issue, we consider using Raman spectra of powder/drop-casted film samples rather than dispersions. Figure 3B shows Raman spectra in the low-frequency RBM range of 150–350 cm^−1^ and performed at an excitation wavelength of 671 nm. This region assists in studying the electronic transitions (m- and s-SWCNT), chirality, and species’ diameter within a given sample. Raman spectrum of the raw SWCNTs in DMF exhibits two dominant RBM features. A broad feature arises in the region 150–225 cm^−1^ attributed to m-SWCNTs, and several peaks in the region 225–350 cm^−1^ arise from s-SWCNTs. These distinctive peaks indicate the electronic nanotubes (m- and s-SWCNTs) in the raw dispersion. RBM peaks observed for the s-SWCNTs in the raw dispersion at 282 and 296 cm^−1^ can be recognized as diameters of 0.89 and 0.94 nm. The Raman spectra of LPE SWCNTs showed a decrease in the peak at 282 cm^−1^ and a nearly flat baseline in the m-SWCNT region. This modification can be attributed to the cholesterol-based polymer’s selectivity for s-SWCNTs with diameters greater than 0.89 nm.

At the same time, it also excluded m-SWCNTs from the dispersion. The recovery of the SWCNTs/DMF Raman spectrum in ppt LPE SWCNTs validates the cholesterol-based polymer’s ability to exclude m-SWCNTs from the dispersion. The peak at 296 cm^−1^ is dramatically suppressed in DLPE SWCNTs. Although this peak can be attributed to small diameter s-SWCNTs, it also can be attributed to fullerene-type impurities [65,66,67]. Below, it will be shown that the latter hypothesis better agrees with further analyses, as the (n,m) of extracted SWCNTs is virtually identical for DLPE and LPE processes. Thus, the ’silent’ Raman spectra observed with DLPE SWCNT indicate that the DLPE process has effectively removed all small-diameter s-SWCNTs and m-SWCNTs from the dispersion. Large s-SWCNTs are not observed, as their RBM vibrations are below the Raman cutoff.

We acknowledge that using a single laser excitation wavelength (671 nm) limits the ability to fully characterize the s-SWCNTs and m-SWCNTs due to resonant effects. While our current study incorporates complementary characterization techniques, such as PL mapping and absorption spectroscopy, we commit to employing multiple laser wavelengths in future studies to provide a more comprehensive analysis of SWCNT selectivity and diameter distributions.

**Figure 4:**
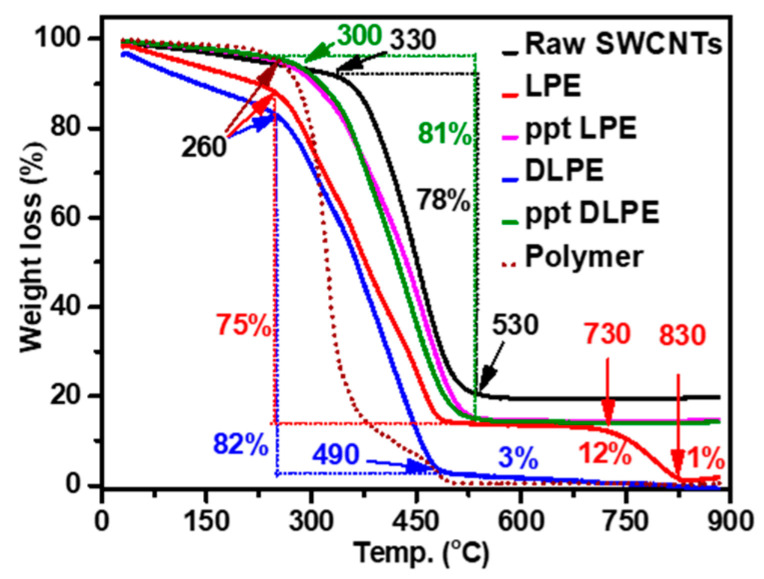
TGA curves (10 °C·min^−1^ under air) of raw HiPCO SWCNTs, poly(CEM_11_-*b*-EHA_7_), LPE SWCNTs, DLPE SWCNTs, ppt LPE SWCNTs, and ppt DLPE SWCNTs.

TGA was used to assess the purity of SWCNT dispersions. When SWCNTs are heated in the air, the mass decreases as the temperature rises due to the materials’ combustion. This mass reduction can be used to determine the quantities of the materials and, hence, evaluate the purity of SWCNTs. As demonstrated in Figure 4, the TGA of raw SWCNTs exhibits two progressive weight decreases. In the first TGA stage, 8 wt% of the raw SWCNTs were decomposed between ambient temperature and 330 °C due to the removal of amorphous carbon [68,69]. The majority (78 wt%) was degraded between 330 °C and 530 °C, which was attributed to the combustion of SWNTs [69,70]. The remainder, representing 20 wt%, corresponds to metallic impurities and high-temperature breakdown components such as nano-graphitic particles. This result reveals that as-grown SWCNTs include 8 wt% amorphous carbon and 20 wt% high-temperature breakdown impurities (metallic and nano-graphitic).

LPE SWCNTs have a decomposition curve that is divided into four temperature regions. First, between ambient temperature and 260 °C, 12 wt% of LPE SWCNTs degraded. The removal of volatile solvents (water and isooctane) explains the larger percentage of weight loss at temperatures below 300 °C compared to the 8 wt% estimated in raw SWCNTs. Following that, 75 wt% of LPE SWCNTs decomposed between 260 °C and 490 °C due to the combustion of both SWCNTs and polymer (CEM_11_-*b*-EHA_7_). The remainder (13 wt%) attained a stable plateau zone of 730 °C before degrading up to 830 °C with 12 wt% loss. The breakdown temperature of 730 °C to 830 °C corresponds to the decomposition temperature of C_60_ fullerene [71,72]. The weight of the remaining material (1 wt%) increased somewhat at temperatures above 800 °C due to the oxidation of residual iron to iron oxides [68,73].

The extracted nanotubes by the DLPE system (DLPE SWCNTs) decomposed in three temperature ranges: 17 wt% decomposed between room temperature and 260 °C due to amorphous carbon and volatile solvents, 82 wt% decomposed between 260 °C and 490 °C due to SWCNTs and poly(CEM_11_-*b*-EHA_7_), and 3 wt% decomposed between 490 °C and 830 °C due to nano-graphitic particles. The continued combustion of DLPE SWCNTs up to 830 °C shows complete removal of metallic impurities utilizing the biphasic technique, which was also efficient in removing most nano-graphitic impurities (3%) compared to 13% in LPE SWCNTs.

In contrast, poly(CEM_11_-*b*-EHA_7_) prefers to exfoliate carbon compounds (SWCNTs, amorphous carbon, and nano-graphitic particles) primarily. DLPE can remove these contaminants and extract primarily SWCNTs from the supernatant. Because raw SWCNTs contain a large amount of residual metallic catalysts and carbonaceous contaminants, the quality of the extracted nanotubes is greatly improved by utilizing the DLPE technique, as confirmed by TGA.

TGA was employed again to analyze the compositions of their precipitates to validate the elimination of metallic impurities from the LPE SWCNTs and DLPE SWCNTs solutions. TGA curves for ppt LPE and ppt DLPE revealed that 4 wt% of the precipitates are volatile solvents and amorphous carbon, which disintegrated at temperatures lower than 300 °C. While SWCNTs account for 81 wt% of the total, they disintegrate between 300 °C and 530 °C. The remaining precipitate (15 wt%) remained undecomposed to 900 °C, demonstrating the precipitation of metallic contaminants in their solutions. During the filtration procedure to collect the precipitates, the 5 wt% difference between the raw SWCNTs and the precipitates (ppt LPE and ppt DLPE) can be lost on the filter paper.

Because the PL is unique to individual and isolated s-SWCNTs [74], PL maps can aid in identifying individualized s-SWCNTs. Still, alterations in the nanotubes’ electronic properties and SWCNT structure induced by isooctane-SWCNT and polymer-SWCNT interactions potentially affect PL intensity, shifted emission wavelengths, or broadened spectral features [64]. To accurately capture the PL signature, careful optimization of acquisition conditions, including appropriate excitation wavelength selection and laser power adjustment, is crucial.

**Figure 5:**
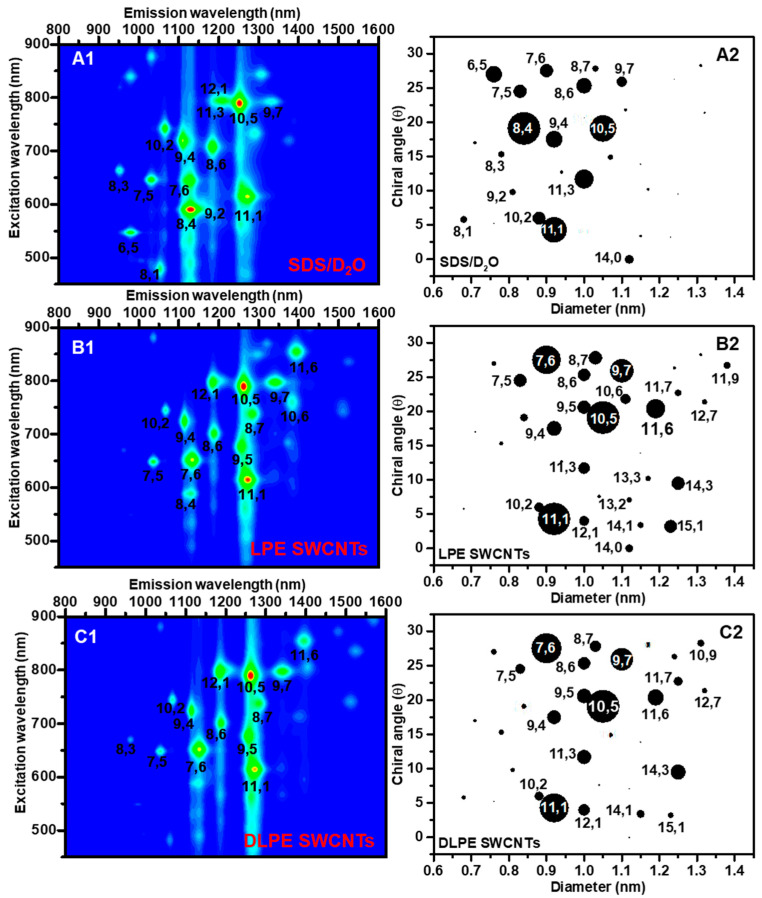
(**A1**–**C1**) are PL excitation−emission maps, and (**A2**–**C2**) θ/d are maps for samples of SDS/SWCNTs, LPE, and DLPE SWCNTs.

As illustrated in Figure 5(A1–C1), the empirical positions for each point in these maps suggest a specific (n,m) indexed tube species. As shown in Figure 5(A2–C2), the chiral angle (θ) was plotted versus the diameter (d) distribution of the extracted s-SWCNTs nanotubes. The obtained PL intensities from PL spectra (Supplementary Figure S1) are proportional to the number of nanotubes in the dispersion. These intensities were directly used to assess nanotubes’ chirality and diameter distributions. The circle area in the θ/d maps represents the abundance of nanotubes observed by the PL analysis. Because of PL quenching by dispersing all types of nanotubes, including m-SWCNTs, residual metallic catalysts, and carbonaceous impurities present in raw SWCNTs, PL mapping for DMF/SWCNT dispersion is complicated [56]. As a result, the PL maps for DLPE SWCNTs and LPE SWCNTs were compared to the PL map for SWCNT dispersion in SDS/D_2_O solution (SWCNTs/SDS).

SDS, a non-selective dispersant, exhibits no apparent chirality preference in Figure 5(A1,A2) [23,75]. The SWCNTs/SDS dispersion included twelve primary semiconducting species with diameters ranging from 0.75 to 1.05 nm: (6,5), (7,5), (7,6), (8,4), (8,6), (8,7), (9,4), (9,7), (10,2), (10,5), (11,1), and (11,3). Significant differences were detected in the (n, m) and diameter size distributions of DLPE and LPE SWCNT samples. These mappings refute s-SWCNT bundle formation and corroborate the efficiency of poly(CEM_11_-*b*-EHA_7_) in individualizing s-SWCNTs. Figure 5(B1,B2) demonstrate that the LPE SWCNTs preferentially disperse five primary types with diameters ranging from 0.9 to 1.1 nm: (7,6), (9,7), (10,5), (11,1), and (11,6). Large-diameter tubes (d = 1–1.3 nm) were also found, such as (14,3) and (15,1). Alternatively, DLPE SWCNTs (Figure 5(C1,C2)) excluded (15,1) nanotubes from the extraction while maintaining the preference for (7,6), (9,7), (10,5), (11,1), (11,6), and (14,3).

Based on the relative abundance of nanotubes, the DLPE technique appears to prefer nanotubes with diameters ranging from 0.9 to 1.1 nm. The similarity of the PL mappings for LPE and DLPE stems from using the identical poly(CEM_11_-*b*-EHA_7_), solvent (i.e., isooctane), and operating parameters for both techniques, except the DLPE technique containing an aqueous phase and does not require any sonication. In comparison to the one-step ATPE technique for HiPCO SWCNTs [58], ATPE demonstrated a preference for diameters below 0.9 nm of (6,5), (7,5), (7,6), (8,3), (8,4), (8,6), (9,4), and (10,4). (10,2). The DLPE technique produces high-purity dispersions in a readily accessible manner with larger diameters.

**Figure 6:**
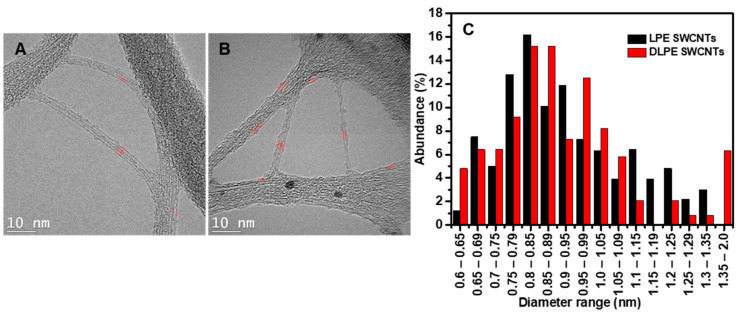
TEM images for (**A**) LPE SWCNTs and (**B**) DLPE SWCNTs. (**C**) The diameter distribution for nanotubes was estimated based on a sampling of ~100 nanotubes in the TEM images for LPE SWCNTs and DLPE SWCNTs. The red lines in images A and B highlight the nanotube edges, marking the regions where diameters were measured from the TEM images.

Using TEM, characteristics corresponding to nanotubes wrapped by a thin layer of poly(CEM_11_-*b*-EHA_7_) can be observed, proving that the polymer might individualize nanotube bundles, as shown in Figure 6A,B. A sample of 100 nanotubes in the TEM images determined a diameter size range of 0.65–1.3 nm (Figure 6C). According to LPE SWCNTs, 50% of the dispersed nanotubes had a diameter range of 0.75–0.95 nm. DLPE SWCNTs revealed that 55% of the extracted nanotubes had larger diameters in the 0.85–1.0 nm range. DLPE can also extract large nanotubes with diameters in the 1.4–2.0 nm range, representing approximately 5% of the nanotubes analyzed in the TEM measurements. Noteworthy, the range from 1.4 to 2.0 nm is represented by 13 distinct intervals within Figure 6C (i.e., 1.4–1.45, 1.45–1.5, 1.5–1.55, and so on up to 2.0 nm). The nanotubes within this range account for approximately 5% of the ~100 nanotubes imaged by TEM. The diameter scale in Figure 5(C2) ends at 1.4 nm. If extended to 2.0 nm, only a few tiny dots would be distributed between 1.4 and 2.0 nm. These dots would be sparse and scattered, making them unlikely to form a distinct circle or cluster representing 5% of the θ/d map.

## 4. Conclusions

Poly(CEM_11_-*b*-EHA_7_), a non-conjugated cholesterol-based polymer, significantly enhanced the enrichment of multi-chirality s-SWCNTs in isooctane. Interestingly, compared to conjugated polymers often used to disperse nanotubes, this polymer is less likely to undergo charge transfer between the nanotube surface and the polymer, making it less urgent to remove the adsorbed polymer for further applications. Still, we acknowledge that the polymer’s presence could still affect the contact quality and performance of CNTs in FETs and similar devices. Further work may be needed to evaluate the impact of the polymer on device performance and explore potential strategies for polymer removal if required. Comparing the LPE and DLPE techniques, we demonstrated that the polymer can selectively extract large-diameter s-SWCNTs from HIPCO SWCNT samples. Small diameter s-SWCNTs, m-SWCNTs, and metallic impurities are thus effectively separated while the DLPE and LPE processes extract (7,6), (9,7), (10,5), (11,1), and (11,6) SWCNTs with diameters ranging from 0.9 to 1.1 nm. Interestingly, the DLPE can also separate the fullerene-type impurities, which remain in the sample with the LPE technique. Such differences may be attributed to the gentler process (the absence of a high-power sonication step) in DLPE, which allows for a true sorting equilibrium to be achieved. Thus, we envision that combining an alkane solvent, the non-conjugated cholesterol polymer, and the DLPE process may produce high-purity, large-diameter s-SWCNT inks.

## Data Availability

We acknowledge the reviewer’s concern and would like to clarify that the data supporting our findings are available and can be provided upon request. We would be happy to share the data to ensure transparency and facilitate further understanding.

## Supplementary Materials

The following supporting information can be downloaded at https://www.mdpi.com/article/10.3390/nano15010023/s1. Supplementary data associated with this article can be found in the online version. Figure S1 shows PL spectra of LPE SWCNTs and DLPE SWCNT dispersions compared to the PL spectra for SWCNT–SDS dispersion.
