# Supplementary material for: Separation of Highly Pure Semiconducting Single-Wall Carbon Nanotubes in Alkane Solvents via Double Liquid-Phase Extraction
Source: Nanomaterials (Basel). 2024 Dec 27;15(1):23. doi: 10.3390/nano15010023 (PMC11721876; doi:10.3390/nano15010023)
Supplement: Supplementary file 1 [file nanomaterials-15-00023-s001.zip › nanomaterials-3375986-supplementary.pdf]

Supporting Information

Separation of Highly Pure Semiconducting Single Walled Carbon Nanotubes in Alkane Solvents via Double Liquid Phase Extraction

Ahmad Al Shboul,*[1] Mohamed Siaj,*[2] and Jerome Claverie,*[3]

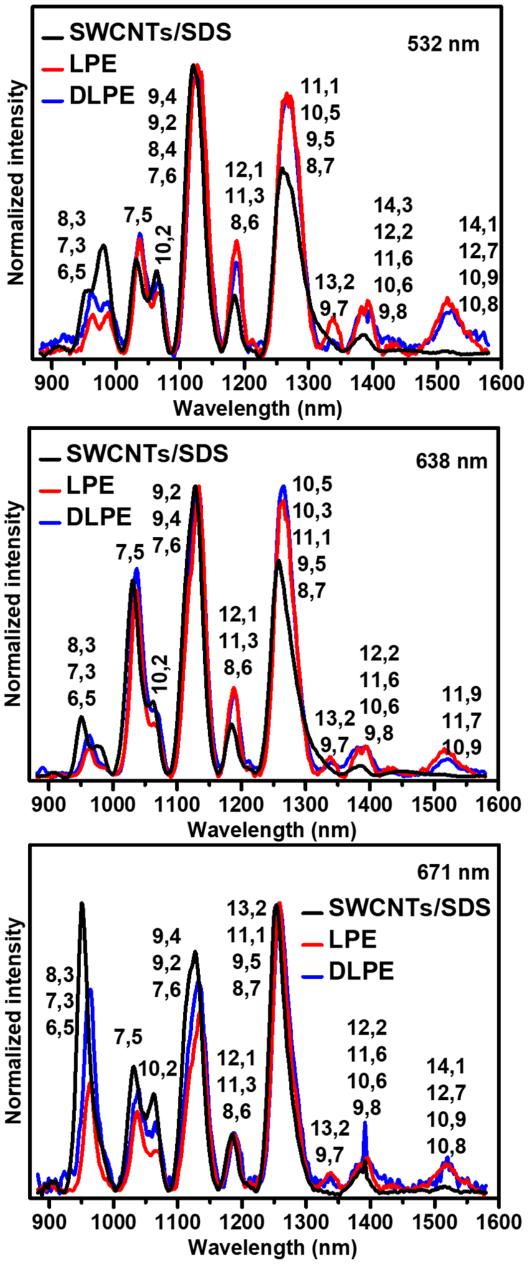

Figure S1. PL spectra of LPE SWCNTs and DLPE SWCNTs dispersions compared to the PL spectra for SWCNT– SDS dispersion. These spectra were produced from laser excitations with wavelengths 532 nm, 638 nm, and 671 nm.
